# CycLing and EducATion (CLEAT): protocol for a single centre randomised controlled trial of a cycling and education intervention versus standard physiotherapy care for the treatment of hip osteoarthritis

**DOI:** 10.1186/s12891-023-06456-0

**Published:** 2023-05-03

**Authors:** Thomas W Wainwright, Erika P Parkinson, Tikki Immins, Sharon Docherty, Elizabeth Goodwin, Annie Hawton, Matthew Low, Joanna Samways, Tim Rees, Geoff Saunders, Robert G Middleton

**Affiliations:** 1 Orthopaedic Research Institute, Bournemouth University, Bournemouth, UK; 2 University Hospitals Dorset NHS Foundation Trust, Bournemouth, UK; 3 TopMD Precision Medicine Ltd, Southampton, UK; 4 Department of Medical Sciences and Public Health, Faculty of Health and Social Sciences, Bournemouth University, Bournemouth, UK; 5 Health Economics Group, Institute of Health Research, University of Exeter Medical School, University of Exeter, Exeter, UK; 6 NIHR Applied Research Collaboration - South West (PenARC), University of Exeter Medical School, University of Exeter, Exeter, UK; 7 NIHR Research Design Service South West, University of Exeter Medical School, University of Exeter, Exeter, UK; 8 Department of Rehabilitation and Sports Sciences, Faculty of Health and Social Sciences, Bournemouth University, Bournemouth, UK; 9 University of Southampton Clinical Trials Unit, Southampton, UK

**Keywords:** Osteoarthritis, Hip, Exercise therapy, Group exercise, Cycling, Patient education

## Abstract

**Background:**

Osteoarthritis (OA) is a chronic degenerative joint disorder for which there is no known cure. Non-surgical management for people with mild-to-moderate hip OA focuses mainly on alleviating pain and maximising function via the National Institute for Health and Care Excellence (NICE) recommended combination of education and advice, exercise, and, where appropriate, weight loss. The CHAIN (Cycling against Hip pAIN) intervention is a group cycling and education intervention conceived as a way of implementing the NICE guidance.

**Methods:**

**C**yc**L**ing and **E**duc**AT**ion (CLEAT) is a pragmatic, two parallel arm, randomised controlled trial comparing CHAIN with standard physiotherapy care for the treatment of mild-to-moderate hip OA. We will recruit 256 participants referred to the local NHS physiotherapy department over a 24-month recruitment period. Participants diagnosed with hip OA according to NICE guidance and meeting the criteria for GP exercise referral will be eligible to participate. Primary outcome is the difference in Hip Disability and Osteoarthritis Outcome Score (HOOS) function, daily living subscale between those receiving CHAIN and standard physiotherapy care. Secondary outcomes include performance-based functional measures (40 m walking, 30s chair stand and stair climb tests), ability for patient to self-care (patient activation measure) and self-reported health-related resource use including primary and secondary care contacts. The primary economic endpoint is the number of quality adjusted life years (QALYs) at 24 weeks follow-up. The study is funded by the National Institute for Health Research, Research for Patient Benefit PB-PG-0816-20033.

**Discussion:**

The literature identifies a lack of high-quality trials which inform on the content and design of education and exercise in the treatment of patients with hip OA and explore cost-effectiveness. CLEAT is a pragmatic trial which seeks to build further evidence of the clinical benefits of the CHAIN intervention compared to standard physiotherapy care within a randomised, controlled trial setting, and examine its cost-effectiveness.

**Trial registration number:**

ISRCTN19778222. Protocol v4.1, 24th October 2022.

**Supplementary Information:**

The online version contains supplementary material available at 10.1186/s12891-023-06456-0.

## Background

Osteoarthritis (OA) is a long-term health condition for which there is no cure. Mechanical and systemic factors may result in joint pain leading to a reduction in quality of life and activities of daily living [1]. Data collected between 2003 and 2010 revealed that around 8.75 million people aged 45 years and over sought treatment for OA in the UK [2]. A 2017 assessment of the prevalence of joint-specific OA revealed that the hip is the second most common joint to be affected after the knee [3]. The Musculoskeletal Calculator estimates that in England 10.9% of people aged 45 years and over have hip OA [2, 4]. Although the risk of developing OA increases with age, a study of trends in incidence of OA between 2012 and 2018 showed a significant increase in the incidence of younger patients with hip OA [5].

Given its prevalence, OA represents a significant economic burden, due to the impact on people’s ability to work and their need for social care and welfare benefits as well as on-going healthcare [6]. According to the National Institute for Health and Care Excellence (NICE) guidelines [7], non-surgical management for people with mild-to-moderate hip OA involves education and advice, exercise (aerobic and local muscle strengthening), and, where appropriate, weight loss. The NICE guidelines do not, however, advise on specific types of exercise, exercise dose, or exercise intensity [7].

A systematic and network meta-analysis review concluded that low impact single exercise interventions, such as aerobic exercise (e.g., swimming, cycling), were the most effective therapies for improving pain and function in hip OA [8]. Indeed, Goh et al. found that therapies consisting of mixed exercises were the least effective in improving pain and function. However, several systematic reviews have highlighted the need for further research into more intense, single-exercise interventions specifically for hip OA [8–11].

A review of potential mechanisms underpinning the benefits of exercise therapy on pain and function in OA found that an increase in upper leg strength, a decrease of extension impairments, and an improvement in proprioception, were all possible mediators of the link between exercise and a decrease in OA symptoms in the lower limb; another review highlighted the need to address muscle weakness in people with hip OA [12, 13]. Cycling has been found to improve balance and proprioception, induce muscle hypertrophy, directly improve muscle weakness and therefore upper leg strength, and (because it is non-weight-bearing) causes less stress on the joints than running or other impact sport [14–16]. Additionally, longitudinal epidemiological studies [17] have shown that cycling can increase cardiorespiratory fitness and functional ability, and lead to significant risk reduction for all-cause and cancer mortality, cardiovascular disease, colon and breast cancer, and obesity morbidity in the middle-aged and elderly.

As a way of implementing the NICE guidelines for hip OA, a cycling and education intervention, the CHAIN (Cycling against Hip pAIN) programme, was developed in 2013 through discussions with local orthopaedic teams and physiotherapists [18] and was updated following feedback from past CHAIN participants. The CHAIN intervention aims to equip participants with the confidence to self-manage their condition, to encourage joint mobility and reduce pain through an 8-week programme of education and static cycling sessions [18–20]. The intervention has been designed to influence behaviour change and includes components to motivate, increase adherence, and reduce drop-out of participants.

### Aim and objectives

The primary clinical objective is to determine whether there is a difference in the change in self-reported function from baseline to post-treatment assessment between those receiving the 8 weeks cycling and education intervention compared to those who received routine physiotherapy care. Self-reported function is assessed using the Hip Disability and Osteoarthritis Outcome Score (HOOS) function, daily living subscale [21]. The primary economic endpoint is the cost per Quality-Adjusted Life Year (QALY) at 3 months post treatment.

The secondary objectives are to estimate cost-effectiveness of the CHAIN intervention by comparing health-resource use of the participants on the two arms of the study through completion of a questionnaire; and to compare changes between the groups in:

1. Self-reported level of hip pain assessed with the Pain subscale of the Hip Disability and Osteoarthritis Outcome Scale (HOOS) [21].
2. Performance assessments: 40 m walking test, 30s chair stand test, Stair Climb test [22].
3. Function level in performing five important activities they have difficulty with, assessed by the Patient-Specific Functional Scale [23] Participants identify up to five important activities they have difficulty performing and rate their current level of difficulty on a scale of 0 to 10.
4. Participant’s knowledge, skill, and confidence to manage their own health assessed using the Patient Activation Measure questionnaire [24].
5. Quality of life assessed with the EQ-5D-5 L and EQ-5D VAS questionnaire [25].

Data will be collected within two weeks of those on the CHAIN intervention arm completing their treatment and collected by post/email or telephone at 3 months post-treatment (with the exception of the objective function assessments).

## Methods

This pragmatic trial follows a successful feasibility study of the intervention [18, 19] and a case study of a participant in that study [26], which demonstrated that patients with complex comorbidities (such as ankylosing spondylitis, hypertension, type 2 diabetes, Crohn’s disease, gallstones, mild asthma, and bronchitis) are able to safely take part and benefit from the programme.

**Fig. 1:**
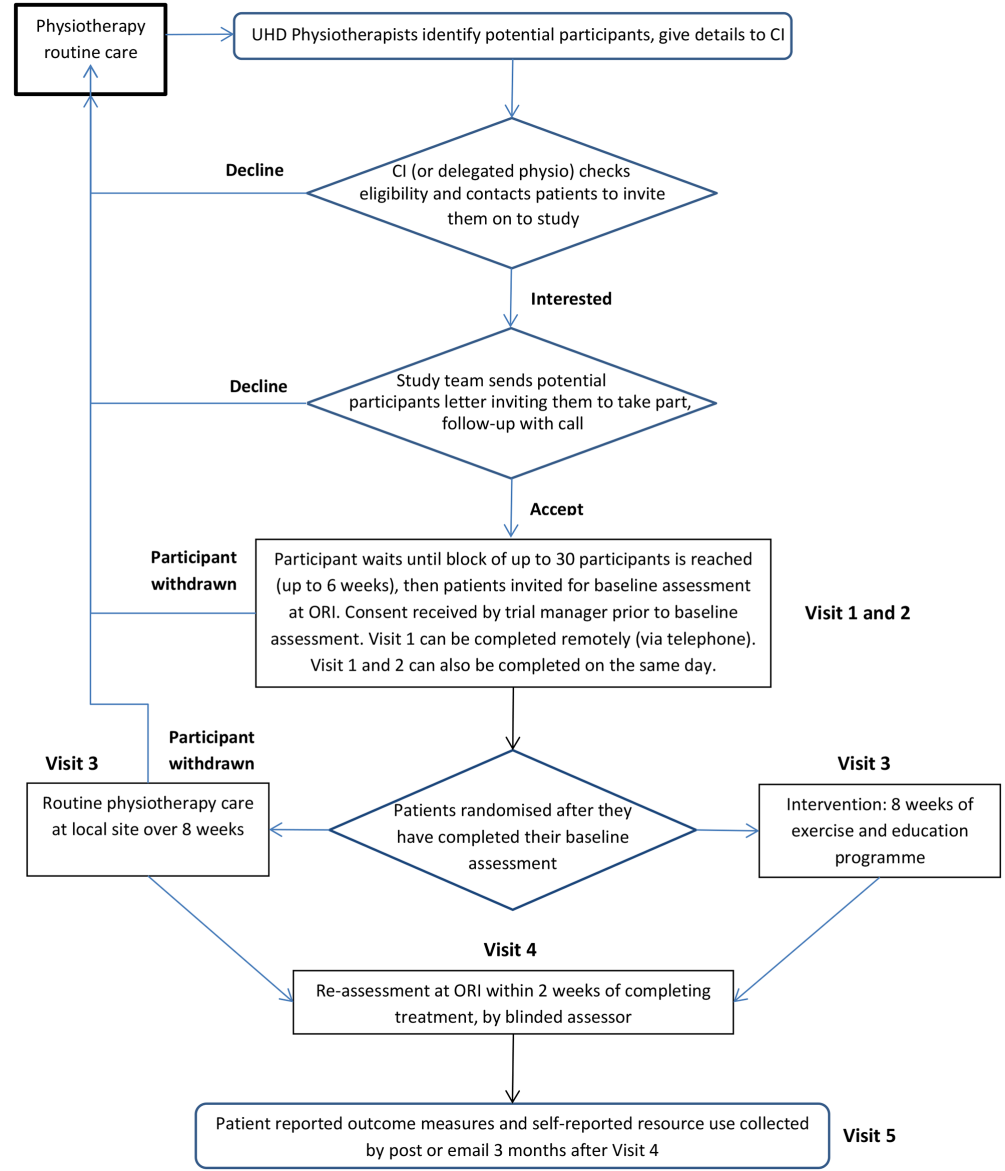
CLEAT Trial flow diagram showing study design

A flow diagram showing the design of this trial is shown in Fig. 1 and the schedule of assessments is presented in Table 1.

### Trial design, setting and oversight

CLEAT is a pragmatic, two parallel arm, randomised controlled trial comparing a cycling and education intervention (CHAIN) with standard physiotherapy care reflecting standard practice in the National Health Service (NHS) in the UK [27]. The protocol has been developed with consideration of the recommendations for the minimum content of a clinical trial protocol as described in the SPIRIT 2013 statement [28].

This trial is open to patients who are referred to the NHS Physiotherapy Department with hip pain. For participants in the intervention arm, the trial will be conducted at a leisure centre, which was involved in the feasibility study. Participants in the (standard physiotherapy care) control arm, will be treated by the NHS Physiotherapy team they have been referred to.

**Table 1:** SPIRIT^*^ diagram of enrolment, treatment, and assessments for the CLEAT study

Timepoint	Visit 1: Verbal consent and pre-baseline	Visits 1 and 2 may be combined Visit 2: Baseline (≤ 2 weeks prior to start of treatment)	Visit 3: Treatment	Visit 4: Post treatment (≤ 2 weeks after end of 8 weeks treatment period)	Visit 5: Long term follow-up (3 months post Visit 4 (± 4 weeks))
**Enrolment:**					
Eligibility screening (including medical history)		X			
Informed Consent	X verbal	X written			
Randomisation		X			
**Interventions**:					
CHAIN			X		
Standard care physiotherapy			X		
**Assessments**:					
Primary outcome					
^**†**^HOOS (function, daily living)		X		X	
Secondary outcomes					
HOOS (function, daily living)		X			X
HOOS (other subscales)		X		X	X
^**‡**^PAM		X		X	X
30-sec sit-stand test		X		X	
Stair climb test		X		X	
40 m walk test		X		X	
^**§**^BMI, % body fat, ^**¶**^BP,		X		X	
resting ^**#**^HR					
Analgesia Use (**RUQ)		X		X	X
Other measures					
Psychological questionnaire and patient feedback (CHAIN arm only)				X	
Interaction with CHAIN video content			X		
Range of motion of hip		X		X	
^**††**^PSFS		X		X	X
Exercise Diary			X		
Adverse Events		X	X	X	
Descriptive measures					
Age, gender, employment status, ethnicity, education		X			
Health economic data					
EQ-5D-5 L		X		X	X
RUQ		X		X	X

*Standard Protocol Items: Recommendations for Interventional Trials (SPIRIT); ^†^Hip Disability and Osteoarthritis Outcome Score (HOOS); ^‡^Patient Activation Measure (PAM); ^§^Body Mass Index (BMI); ^¶^Blood Pressure (BP); ^#^Heart Rate (HR); **Resource Use Questionnaire (RUQ); ^††^Patient-Specific Functional Scale (PSFS)

### Participants

We intend to recruit participants with hip pain or hip OA. OA will be diagnosed by a clinician using the NICE criteria [29] to review medical history and current symptoms. NICE recommend that OA is diagnosed if a person is 45 or over; has activity-related joint pain; and has either no morning stiffness or morning stiffness that lasts no longer than 30 min. An x-ray will not be required to confirm the diagnosis unless the participant is aged under 45. Eligibility criteria can be found in Table 2.

**Table 2:** Eligibility Criteria for the CLEAT study

Inclusion Criteria	Exclusion Criteria
Male and female aged 18 years and over. If under 45, an x-ray confirming diagnosis of osteoarthritis is required. *	Hip surgery within the last 6 months
Diagnosed with osteoarthritis of the hip as per NICE criteria	On the waiting list for a hip replacement or planning back or lower limb surgery in the next 9 months
Meeting the GP criteria for exercise referral (British Heart Foundation 2010)	Current or past (within 3 months) intra-articular corticosteroid injection (or other injection) of the hip*
Capable of giving informed consent	Women who are pregnant and have not previously or are not currently exercising regularly to the equivalent of 30 min of static cycling per week
Willing to commit to the exercise intervention if randomised to the treatment arm	Judged by investigator to have high levels of functional limitations which will prevent the participant from getting on and off the exercise bike
Able to commit to the exercise intervention if randomised to the treatment arm as assessed by the physiotherapist after reviewing participant medical records at the baseline assessment	Due to the safety limitations of static bikes used, participants need to be ≥ 150 cm tall and weight ≤ 135 kg.
Be able to understand English as necessary to benefit from the intervention, in the investigator’s opinion	

*Eligibility criteria amended (Amendment 2, 25th April 2021 and Amendment 4, 7th October 2022) after discussion with the Trial Steering Committee to increase recruitment rates. Previously participants had to be aged 45 or over and were ineligible if had current or past (within 3 months) oral or intra-articular corticosteroid use

### Recruitment

Potential participants will be identified from Physiotherapy waiting lists by senior physiotherapists and screened by the research team as per exclusion criteria (Table 2). Eligible participants will be contacted by telephone (or by letter if unable to contact by telephone) by a senior physiotherapist to introduce the study. If interested in receiving more information, an invitation letter and participant information sheet (see supplementary files 1 and 2), will be sent by post. Eligible participants will receive a further telephone call a few days later to discuss the trial and answer any questions. If the participant is interested, they will be added to a waiting list. Once up to 30 participants are on the waiting list they will be invited for final screening and baseline assessment. Physiotherapy referral rates indicate that it will take less than 10 weeks to recruit 30 participants, which is less than the current waiting time for routine appointments at the trial site (currently 52 weeks).

### Pre-baseline and baseline (visits 1 and 2)

Prior to the baseline visit, the Trial Manager will contact participants by telephone to discuss the trial, offer participation and receive verbal informed consent (supplementary file 3). It will be explained that participation is voluntary, and they can withdraw at any time. During the telephone appointment, once verbal consent has been received, baseline data collection can commence.

Baseline assessments (Visit 2) will take place on the same day as randomisation. The participant will be asked to sign the informed consent form by the trial manager, or a delegated member of the research team, and the remaining baseline data will be collected by the trial assessors (see the Outcome measures section) and will include a review of participants’ medical history; physical measurements such as height, weight, body composition (measured with an electronic segmental body composition monitor (Tanita MC-780MA Multi Frequency Body Composition Analyser https://fitech.uk/medical-class-scale-systems/47-tanita-mc-780ma-s-multi-frequency-body-comp-analyser-portable.html), blood pressure, resting heart rate and range of motion of both hips. Performance assessments as recommended by the Osteoarthritis Research Society International (OARSI) for core performance tests will be collected [22] :30 s chair stand test, stair climb test and 40 m walk test. Patient reported outcome measures will be collected through validated questionnaires: the HOOS [21]; the EQ-5D-5 L and EQ-5D VAS [25]; the Patient-Specific Functional Scale [23] and the Patient Activation Measure [24}.

Assessments will be undertaken by qualified independent physiotherapists who have been trained by the trial investigators. A summary of the data to be collected at Baseline can be found in Table 1.

As part of baseline assessments, participants will also be asked to do an exercise tolerance test which involves riding a static cycle for up to 18 min, with heart rate, blood pressure and exertion rates tested at intervals. Any participant outside the expected parameters for these tests and judged by the physiotherapist assessors to be unfit for the cycling intervention, or anyone not wishing to take part in the cycling and education intervention, will not continue the study. Participants are enrolled on the study after baseline assessments have been completed and they have been randomised to receive either the CHAIN intervention or standard physiotherapy treatment. It is possible for Visit 1 and Visit 2 to be carried out on the same day as part of the baseline assessment. Treatment will commence within the following two weeks for both arms.

### Randomisation

Participants will be randomised using permuted blocks of size 2, 4 or 6 with parallel group randomisation in a 1:1 ratio to either CHAIN or standard physiotherapy treatment. Ideally the CHAIN intervention is run with 15 participants, so recruitment will be based on blocks of 30 eligible participants. However, the programme can proceed with fewer participants if necessary to prevent undue delays in treatment. With the use of block randomisation, we hope to achieve equal numbers of participants in each arm of the trial while maintaining treatment allocation concealment from the trial assessors.

A list of randomised allocations will be created for the whole study prior to the first person being recruited.

Once consent has been received and screening/baseline assessments completed, the trial manager (or delegated person) will randomise the participant, using the randomisation system incorporated within the trial database, to receive the next treatment allocation from the randomised list. If a participant does not complete the screening/baseline procedures or meet the required eligibility criteria, they will not be randomised and will be removed from further involvement with the study.

Assessors undertaking assessments at baseline and follow-up will be blinded to the randomisation, and participants will be educated to ensure that they do not inform the assessors of which treatment arm they participated in. The nature of the intervention means that participants and treatment providers will not be blinded.

### Intervention (visit 3)

Treatment will commence within 2 weeks of randomisation at Baseline Assessment.

### 8 Weeks cycling and education programme (CHAIN)

For 8 weeks following randomisation, participants in groups of up to 15, will attend a weekly, one hour education and cycling session at a local leisure centre. Each session will typically involve a 30 min education session which aims to motivate participants to exercise, reassure them that it is safe to do so, and facilitate positive lifestyle/activity change, followed by a 30 min indoor static cycling class (35 and 40 min for the last two sessions). An overview of CHAIN week-by-week is shown in Fig. 2, and a full description of this intervention following TIDIER guidelines [30] can be found in Table 3.

**Fig. 2:** Overview of the content for the CHAIN intervention, week-by-week

Week	Week 1	Week 2	Week 3	Week 4	Week 5	Week 6	Week 7	Week 8
Education session (30 min)	Introduction	Review of last session	Review of last session and activity diary	Review of last session and activity diary	Review of last session and activity diary	Review of last session and activity diary	Review of last session and activity diary	Review of last session and activity diary
	Aims of programme	Introduction to activity diary	Complementary therapies	Pacing	Nutraceuticals and supplements	Oral analgesics	Self-management tips and planning	Summary of previous sessions
	Introduction to osteoarthritis (OA) and the hip joint	Introduction to Home Exercise Programme (HEP)	Assistive devices and footwear	Manual therapy, manipulation and stretching	Alternative exercise options	Topical treatments	Injections and surgical options Part I	Injections and surgical options Part II
	Benefits of exercise for OA Part I	Benefits of exercise for OA Part II	Diet, nutrition and weight loss	Thermotherapy	Electrotherapy and acupuncture	Non-steroidal anti-inflammatory drugs (NSAIDs)	Post programme exercise planning	Post programme exercise and support networks
	Cycling technique Part I	Cycling technique Part II	Cycling technique Part III	Cycling technique Part IV	Cycling technique Part V	Cycling technique Part VI	Cycling technique Part VII	Cycling technique revision
Static Cycling Session	30 min	30 min	30 min	30 min	30 min	30 min	35 min	40 min
Home Exercise Programme (HEP)	Daily HEP 2 × 30 min cycling per week	Daily HEP 2 × 30 min cycling per week	Daily HEP 2 × 30 min cycling per week	Daily HEP 2 × 30 min cycling per week	Daily HEP 2 × 30 min cycling per week	Daily HEP 2 × 30 min cycling per week	Daily HEP 2 × 30 min cycling per week	Daily HEP 2 × 30 min cycling per week

**Table 3:** CHAIN Intervention description following the TIDIER checklist [24]

1. Name	CHAIN: Cycling against Hip Pain
2. Why	The rationale for the CHAIN intervention is based on the need to develop an effective model of care locally to deliver the nonsurgical interventions recommended by NICE (2014). It was conceived by a consultant orthopaedic surgeon and a physiotherapist in response to discussions locally with primary care providers suggesting that ‘standard care’ for patients reporting hip stiffness to their GP can be varied and inconsistent, ranging from general advice, advice on analgesia and/or physiotherapy and self -management. It was further developed through discussion with local orthopaedic teams and physiotherapists and in partnership with the local general hospital, the county commissioning group, general practitioner localities, the county sports partnership, the borough council and the university. The aim of the programme is to reduce pain and encourage mobility through a programme of education and static cycling sessions to equip participants with the confidence to self-manage their condition.
3. What: Materials	CHAIN videos which will be shown to participants by the facilitator during the CHAIN sessions and can be accessed by the participants in their own time after the sessions. The cycling sessions were developed by senior physiotherapist and fitness instructors from the leisure centre to ensure that the sessions are the same in each cohort. Figure 2 shows a breakdown of the cycling and education content week-by-week.
4. What: Procedures	**Screening**: Prior to enrolment, participants are reviewed under the GP referral for exercise to ensure their suitability to attend the sessions. **Education**: For the first thirty minutes, participants will take part in an education class, facilitated by a qualified physiotherapist. The education sessions will be standardised through video recordings developed by the study team and based upon NICE guidelines, aiming to promote the effective on-going self-management of hip pain. The education classes also aim to motivate participants to exercise, reassure them that it is safe to do so, and facilitate positive lifestyle/activity change. At the end of the class, the physiotherapist will encourage group discussion so that participants can share their questions and experiences. **Cycling**: The education session will be followed by a 30 min indoor static cycling class (35 and 40 min for the last two sessions respectively), facilitated by a gym instructor trained in leading indoor cycling classes. On the first week, participants will be shown how to set up their bike. The intensity of the exercise class will increase on a weekly basis and will be clearly defined to ensure each cohort will be given the same programme. Each session will finish with a cool-down period which will include relevant stretches. Participants will be encouraged to work at a level that they are comfortable with and will be encouraged to increase their intensity progressively over the eight sessions. **Between sessions**: After completion of the class, the participants will receive a video of the education class and of the static cycling session, via text or email, to encourage exercise and compliance to behaviour change advice at home. A home exercise programme comprising of various ankle, knee and hip stretches will also be provided and participants will be encouraged to stretch regularly. Cycling between sessions will be encouraged but will not be mandatory. To encourage participants to increase their exercise activities, an activity diary will be provided so that progress at home can be recorded and monitored for the duration of the intervention, including planned pauses in the delivery of the intervention. Plans for lifestyle changes and on-going participation in community-based activities will also be discussed.
5. Who provided	**Physiotherapists**: Local NHS senior physiotherapists will facilitate group discussions during each weekly education session. As trained by the study team, they will encourage participants to share experiences and ask questions related to that week’s topic or the management of their hip pain in general. The physiotherapists will also ensure adherence to the intervention by reminding participants to complete their activity diaries in between sessions. **Fitness Instructor**: a qualified and experienced fitness instructor from the local leisure centre will deliver the cycling session each week. The fitness instructors have experience of leading cycling sessions with mixed ability groups and have helped to design the content of each weeks’ session alongside the study team.
6. How	The intervention will be delivered face-to-face in a group setting. After each weeks’ session, participants will be sent a link (via email or text message) to that weeks’ education and cycling session as well as the home exercise programme so that they can be reviewed in between sessions in participants’ own time. A short report detailing changes in assessment scores will be sent to participants following treatment.
7. Where	Intervention will be delivered at a suitable local leisure centre, equipped with space to deliver the education session and an appropriately equipped cycling studio that has space for up to 15 participants to take part in a single cycling session
8. When and how much	The CHAIN intervention is an 8-weeks cycling and education programme where participants will attend a one-hour education and exercise session on a weekly basis at a local leisure centre. It is not necessary for the intervention to be delivered in consecutive weeks and a short pause in delivery of the programme will be possible in the case of local facilities having to close, i.e., due to public holidays or other situations out of the study team’s control. However, if a participant misses a planned course, there will not be an opportunity to provide a catch-up session and the participant will be marked as absent from the session. Dosage received for each participant will be calculated based on sessions attended and length of those cycling sessions along with the recommended cycling intensity.
9. Tailoring	Static cycling is an accessible form of exercise which can be tailored to all levels of ability and fitness within a group setting.
10. Modifications	N/A
11. How well: Planned	Adherence will be assessed by registering the attendance of each participant at each session. Activity diaries will be given to participants to record any activities undertaken outside of the intervention. Participants will be reminded by the senior physiotherapist each week of the importance of the completion of these diaries including recording of the how frequently participants complete the Home Exercise Programme (HEP). The activity diaries will be collected at the Visit 4 and the data analysed, including adherence to the HEP
12. How well: Actual	N/A

### Control arm: standard physiotherapy care

The Trust Physiotherapy Department will allocate treatment slots (within two weeks of randomisation) for participants in the control arm. Once randomised to this arm, at their baseline visit participants are given the next available date and time for their first treatment session. Thereafter the physiotherapy appointments are made between the participant and the Physiotherapy Department directly as per standard care. Over 8 weeks, participants in the control group will attend up to four one-to-one sessions of physiotherapy (as per NHS standard care [27]) for up to two hours. These visits may be face-to-face at the Trust Physiotherapy Department or by telephone depending on the physiotherapist’s standard care practice at the time of treatment. Treatment will be pragmatic and multimodal and may include exercise, education, manual therapy, and other physiotherapy techniques. Participants will also be encouraged to complete a series of home exercises as prescribed by their physiotherapist. The exact treatment received, and the duration and number of sessions delivered will be recorded by the physiotherapist providing the treatment in patient notes and the study proforma (Supplementary File 4). This will enable the dosage of the intervention (type, intensity, and time) for participants in the control arm to be compared to that received by participants in the CHAIN arm. This arm will also complete activity diaries to record activities undertaken outside the physiotherapy sessions and physiotherapists will remind participants of their completion. It will be collected at the post-treatment Visit 4. In instances where it is not possible for the physiotherapy department to conduct all the required appointments within the 8 weeks treatment period (e.g. due to illness or capacity issues) an extension to complete appointments will be possible. However, participants will continue to be assessed within two weeks of the allocated 8-weeks study treatment period, and again 3 months later as per the protocol. Any treatment in the control arm outside of the 8 weeks period will be recorded as such.

### Follow-up (visit 4, visit 5)

There will be two follow up assessments, the first within 2 weeks of the end of the CHAIN intervention (or end of 8 weeks treatment period for the control arm) (Visit 4) and the second (Visit 5) occurring approximately 3 months after the post-treatment visit. Visit 4 will be undertaken by trained, qualified physiotherapists and will consist of the same physical and functional assessments performed at Visit 2, completion of patient questionnaires as well as a review of participants’ activity diaries and a feedback questionnaire for those participants that were randomised to the CHAIN intervention; Visit 5 consists of 5 patient questionnaires (Hip Disability and Osteoarthritis Outcome Score (HOOS); Patient Specific Functional Scale (PSFS); Patient Activation Measure (PAM); EQ-5D-5 L; and resource use) sent to participants by email or post. Participants will be contacted by text/telephone/email to help ensure completion and return of questionnaires. Where this is difficult to achieve, we will prioritise collection of the primary outcome at Visit 4. The data to be collected at each of these follow-up appointments are summarised in Table 1.

### Outcomes

#### Primary outcome

The primary outcome is the function, daily living component from the Hip Disability and Osteoarthritis Outcome Scale (HOOS) [25], measured at Visit 2 and Visit 4.

#### Secondary outcomes

We are measuring a number of secondary outcomes including participant reported outcome measures, resource use and QALYs at both Visit 4 and visit 5; and changes in physical and functional measurements at Visit 4. These are summarised in the SPIRIT diagram (Table 1), but in brief are as follows:

- HOOS function, daily living score measured out of 100, after completion of the HOOS Questionnaire at Visit 2 and Visit 5 (score at Visit 4 is primary outcome);
- HOOS pain score measured at Visits 2, 4 and 5; HOOS symptoms score measured at Visits 2, 4 and 5;
- HOOS stiffness score measured at Visits 2, 4 and 5;
- HOOS sports and recreational activities score measured at Visits 2, 4 and 5;
- HOOS Quality of Life score measured at Visits 2, 4 and 5;
- Function assessed by 30-second chair stand test measured at Visit 2 and 4;
- Function assessed by stair climb test measured at Visit 2 and 4;
- Function assessed by 40-metre walk test measured at Visit 2 and 4;
- Patient activation measured by responses to PAM Questionnaire at Visits 2, 4 and 5;
- BMI, body composition, blood pressure and resting heart rate measured at Visits 2 and 4;
- Analgesia use collected from the Resource Use Questionnaire during Visits 2, 4 and 5;
- Patient-perceived general health measured using EQ-5D Visual Analogue Score (VAS) at Visits 2, 4 and 5;
- Patient-perceived quality of life measured by EQ-5D-5 L Questionnaire at Visits 2, 4 and 5, used to derive Quality Adjusted Life Years (QALYs).
- Self-reported resource use measured by responses to the Resource Use Questionnaire during Visits 2, 4 and 5.

### Adverse events

Hip OA is a non-life-threatening condition typified by on-going progression of symptoms and deterioration of function. From the time the participant signs the consent form until the day the participant has completed their post-treatment assessment (Visit 4), the only adverse events recorded will be those for which participants seek advice from a health care professional, such as an unplanned visit to a General Practitioner for an acute condition. Participants will be asked at the start of each CHAIN session or physiotherapy session whether they have had any adverse events, and they will be asked again at the assessment post-treatment.

Based on our feasibility study [18] as well as a case study of a participant with a number of co-morbidities [26], the intervention is deemed low risk. Therefore, we are not expecting any serious adverse events.

### Sample size calculation

The sample size calculation is based on the primary outcome measure, the physical function score from the HOOS function scale at Visit 4. This has identical items to the Western Ontario and McMaster Universities Osteoarthritis Index (WOMAC) function scale [31], with total score transformed to a percentage. Mean primary outcome will be compared between the intervention and control arms using baseline function score as a covariate. The literature mostly uses the WOMAC function score, so parameters for the sample size calculation are based on that, though we indicate the equivalent HOOS score where relevant. We will assume a correlation between pre and post exercise outcomes of 0.6 and take this into account in the calculation [32]. This is a conservative estimate based on (a) our feasibility study data and Wallis *et al’s* study [33], for which the lower limits of the 95% confidence interval were 0.75 and 0.69 respectively, and (b) Bennell et al.’s research [34] that assumed a value of 0.6 for their sample size calculation. This will reduce the required sample size by a factor of 0.64. To allow for any clustering effects resulting from the group delivery of the CHAIN intervention, we have inflated the variance of this arm by a factor of 1.22, assuming a cluster size of 12 and intra-cluster correlation coefficient of 0.02 [35]. Using a 5% two-sided significance test, an effect size (average of minimum clinically important difference found previously [36, 37]) of 5 points on WOMAC (equivalent to 7.4% on HOOS) and standard deviation (average taken from previous literature [36–39] and the feasibility study [18]) of 13 on WOMAC (equivalent to 19.1 on HOOS) a sample size of 102 per arm would be required for 90% power. Allowing for a withdrawal and/or incomplete primary outcome data rate of 20% (for example [40]) the recruitment target will be increased to 256 (9 clusters of size 14–15 in the intervention arm). The sample size calculation was partly conducted using NQuery Advisor software (San Diego, USA).

### Statistical analysis

Prior to completing data collection, a detailed Statistical Analysis Plan will be written and approved by the Trial Steering Committee. Participants’ data will be analysed in the group they were randomised to, and we will attempt to collect outcome data on all participants unless they requested to be withdrawn from the study (‘intention to treat’ analysis). Analysis and initial write-up will be blinded to treatment groups and reported according to the Consolidated Standards of Reporting Trials. Baseline characteristics of the sample in each arm of the trial (e.g., demographics, body composition, etc.) will be summarised using descriptive methods. The primary outcome of the function component of HOOS measured at Visit 4 will be compared between trial arms using a multi-level/mixed model approach, considering baseline function and CHAIN group (cluster).

We will conduct a per protocol analysis where participants will be excluded from the analysis if (a) there was a protocol violation (including swapping treatment arms) or (b) adherence to exercise was poor (e.g., attendance at less than 87.5% of classes i.e. less than 7 classes) or adherence to physiotherapy was poor in the control arm (e.g. attendance at less than 100% of sessions i.e. participant must attend all physiotherapy sessions recommended by therapist).

### Economic evaluation

The economic evaluation will use data collected within the trial to establish the resources required to provide the intervention, estimate its cost, and provide a full cost-effectiveness analysis (CEA). In order to comply with NICE guidance [41] the economic evaluation will take a primary perspective of the NHS/Personal Social Services, i.e., it will include costs borne by the NHS and statutory social care services only. The impact on cost-effectiveness results of taking participant and societal perspectives, which incorporate costs to patients, their families and wider society, will be explored in sensitivity analyses.

Cost and outcome data will be synthesised to present incremental cost-effectiveness ratios (ICERs) - the incremental cost per unit of additional outcome - for the primary outcome (cost per unit change on the HOOS measure) and the primary economic endpoint of policy relevance (cost per QALY).

Intervention resource requirements and costs (e.g., staff time, venue hire, educational materials, travel, communication, consumables) will be collected at an individual participant level, using case report forms. Resource use data (including primary and secondary healthcare, social care, and participant and carer-related resource use) will be collected at Visits 1/2, 4 and 5, using a self-report Resource Use Questionnaire. This questionnaire will draw on measures used with similar populations in the Database of Instruments for Resource Use Management (DIRUM) repository [42, 43], alongside current practice and recent methodological developments [44] and be honed to this specific population through discussion with the study Patient Advisory Group. QALYs will be derived from EQ-5D-5 L trial data, collected at Visits 1/2, 4 and 5. In line with NICE recommendations [45], EQ-5D-5 L data will be mapped to the EQ-5D-3 L UK tariff [46] using the approved ‘cross-walk’ algorithm [47]. These mapped values will be used to estimate QALYs through application of standard area-under-the-curve methods [48] using Visit 1/2, 4 and 5 assessments. Incremental costs and incremental Quality-adjusted life years (QALYs) over 24 weeks will be used to estimate the cost-per-QALY of CHAIN versus usual physiotherapy care.

We will follow the internationally recognised Consolidated Health Economic Evaluation Reporting Standards (CHEERS) guidelines for reporting cost-effectiveness studies [49]. Descriptive statistics will be used to summarise costs (by type of service) and QALYs. Regression analyses will be used to adjust for any systematic differences between intervention and control arms at baseline that have not been accounted for by randomisation. If required, we will use multiple imputation to correct for bias that may result from data missing at random [50]. Sensitivity analyses will explore uncertainty, and a clear, policy-relevant presentation of findings will be provided. All analyses will be conducted in line with the statistical analysis plan.

A cost-consequences analysis will also present key costs and outcomes in a disaggregated, tabular format [51, 52]. This will enable assessment of the component parts of the intervention, health/social care and patient resource use and costs of care, and multiple outcomes, for the intervention and control arms.

### Patient and participant involvement

A public and participant involvement forum, comprising six people who had undertaken the pilot CHAIN programme has informed this protocol, providing feedback on the study design and documentation, the design of the intervention, the main outcome of interest and how the data would be disseminated [53]. The forum was held in April 2014 at the leisure centre where the pilot programme took place and its findings informed the investigators’ decision to increase the number of sessions in the programme from six to eight weeks, allow further time for questions, and continue to include an exercise diary. The trial Patient Advisory Group (PAG) provided feedback on the Participant Information Sheet (PIS) and the resource use questionnaire. They will provide on-going advice to the Trial Management Group on management of the research, further participant-facing documentation and participant questionnaires, analysis of results and dissemination of findings. The PAG will also identify a member of their group to represent them on the Trial Steering Committee.

### Management and oversight of the study

The sponsor for this study is the University Hospitals Dorset NHS Foundation Trust, and they have overall responsibility for the initiation and management of the trial. The Chief Investigator will be responsible for trial design, data analysis and interpretation, manuscript writing and dissemination. The Trial Management Group will meet approximately 4 times per year to ensure all practical details of the trial are progressing and working well. The Trial Steering Committee will conduct oversight of the study and will meet at least yearly. It will comprise an independent Chair, independent statistician, and member of the Patient Advisory Group, along with the Chief Investigator (CI) and a member of the Sponsor team. As this is a low-risk intervention and there are no plans to undertake interim analysis, the Trial Steering Committee will also act as the Data Monitoring Committee in this study.

All data collected in this trial will be entered into a secure cloud based independently run trial database. All data will be anonymised, and participants given a unique trial number. The research team will be responsible for capturing relevant data onto paper source documents and entering the data into the electronic case report form. Results will be disseminated through open-access peer-reviewed publications, presented at relevant conferences and on our CLEAT website https://www.uhd.nhs.uk/directory/name/28-services/bournemouth/1480-cycling-and-education-cleat .

### Ethics and dissemination

This trial will be conducted in accordance with Good Clinical Practice principles and guidelines, the Declaration of Helsinki, University Hospitals Dorset NHS Foundation Trust standard operating procedures, relevant UK legislation and the trial protocol. Ethical approval was granted on the 5th Nov 2019 (19/SC/0502), by the South Central Oxford C Research Ethics Committee (current approved protocol v4.1, 24th October 202214.1 ). The trial will be reported in line with the CONSORT statement [30, 54] and TIDieR Checklist (for reporting Interventions) [30].

Information about study patients will be kept confidential and managed in accordance with data protection legislation, the UK Policy Framework for Health and Social Care Research and Research Ethics Committee Approval.

Information enabling direct/immediate identification (name, contact details, date of birth) will only be accessible to a limited number of personnel within the research team and will only be used where necessary for the purposes of the study or safeguarding participants. No personal identifiable information will be recorded on any data collection documentation. Only authorized members of the research team will have access to the research data, and any data shared from the participants’ medical records, and patients will be anonymised with regards to any future publications relating to this study. All research data will be stored securely in adherence with the data protection legislation in force, the Sponsor’s data management policies, and the patient information sheet. Consent will be obtained to allow authorised staff employed by the Sponsor to review identifiable data to ensure the study is monitored / audited to assess compliance with the protocol.

## Discussion

The Cochrane Collaboration’s 2014 review of studies of exercise for hip OA found evidence supporting the use of exercise to reduce pain and improve physical function [55]. Furthermore, treatment of early OA of the knee and hip with exercise has been shown to have cost benefits [56–58]. However, the most recent systematic review of physical therapy interventions for hip or knee OA resulted in only 9 eligible studies that included economic analysis, highlighting the need for more high-quality studies which can inform future clinical practice [59]. The CLEAT trial seeks to build further evidence of the clinical and cost effectiveness of the CHAIN intervention compared to standard physiotherapy care within a randomised, controlled trial setting.

This trial aims to generate findings that are directly applicable to routine hospital care, and as such the standard care arm is pragmatic representing a mixture of approaches in the treatment of OA [27]. To compare the effectiveness of CHAIN versus standard care, the dosage of treatment given, in terms of type, intensity, and time, will be collected. Additionally, this will allow the comparison of the dose response of this study with other exercise interventions. Our inclusion criteria are broad, allowing for a wide range of participants to be recruited. For example, we will only exclude participants who have had hip surgery within 6 months of recruitment to the trial. The CHAIN intervention has been evaluated with a large number of participants previously and it has been shown that participants with multiple co-morbidities can safely take part in this type of intervention [18, 20, 26]. All participants will be encouraged to take part in additional exercise which will be recorded in trial exercise diaries with the aim to compare the type and duration of exercise undertaken between the two trial arms.

The trial takes place at a single high-volume site; this is mainly due to reducing the burden of travel for the participants in the CHAIN arm because the intervention will be delivered at a leisure centre with previous experience of hosting CHAIN, and to simplify the operational running of the study. Dorset Council has one of the oldest populations in the United Kingdom (29.6% over 65 years compared to the national average of 18.6% [60]), and the University Hospitals Dorset NHS Foundation Trust has some of the highest rates of hip surgery in the country (1091 primary hip replacements in Dorset NHS hospitals in 2019, 367 in independent hospitals [61]), thus making Dorset an ideal location for recruitment. A limitation to this trial is the limited long-term follow up, and therefore, determining the effect of the intervention on the time to surgery and other related disease specific health care costs beyond 3 months after treatment finishes will not be possible. We intend to seek further funding for longer term follow up of these trial participants in the coming months.

### Trial status

Participant recruitment commenced on 29th January 2020; however recruitment was paused until June 2021 due to the global pandemic, and participants recruited before the pandemic had to be withdrawn as they had not completed treatment. Recruitment should be completed by May 2023 and completion of the study by Spring 2024.

To date, there have been three substantial amendments:

Amendment 1 9th June 2020. Changes to study patient documentation due to pause during Covid pandemic.

Amendment 2 25th April 2021. Changes made to study timings and location due to Covid restrictions, extra demographics collected, exclusion criteria on steroid use adapted to omit use for asthma.

Amendment 4 7th October 2022. Exclusion criteria on age and steroid use updated to increase recruitment, study patient documentation updated to include report sent to CHAIN participants on change in outcomes, and leaflets giving participants details on where to attend treatment.

## Electronic supplementary material

Below is the link to the electronic supplementary material.

Supplementary Material 1

Supplementary Material 2

Supplementary Material 3

Supplementary Material 4

Supplementary Material 5

## Data Availability

Once the research has been completed, the data will be made freely available. The dataset analysed in the study will be made available from the corresponding author on reasonable request.

## Abbreviations

BP: Blood Pressure
BMI: Body Mass Index
CEA: Cost-effectiveness Analysis
CHAIN: Cycling against Hip Pain programme (intervention)
CHEERS: Consolidated Health Economics Evaluation Reporting Standards
CLEAT: Cycling against Hip pAIN (study name)
DIRUM: Database of Instruments for Resource Use Management
HEP: Home Exercise Programme
HOOS: Hip Disability and Osteoarthritis Outcome Score
HR: Heart rate
ICER: Incremental cost-effectiveness ratio
NICE: National Institute for Health and Care Excellence
NHS: National Health Service
OA: Osteoarthritis
OARSI: Osteoarthritis Research Society International
PAM: Patient Activation Measure
PAG: Patient Advisory Group
PIS: Participant information sheet
PSFS: Patient Specific Functional Scale
QALY: Quality-Adjusted Life Year
RfPB: Research for Patient Benefit
RUQ: Resource Use Questionnaire
SPIRIT: Standard Protocol Items: Recommendations for Interventional Trials
UK: United Kingdom
VAS: Visual Analogue Scale
WOMAC: Western Ontario and McMaster Universities Osteoarthritis Index
