# Supplementary material for: CycLing and EducATion (CLEAT): protocol for a single centre randomised controlled trial of a cycling and education intervention versus standard physiotherapy care for the treatment of hip osteoarthritis
Source: BMC Musculoskelet Disord. 2023 May 3;24:344. doi: 10.1186/s12891-023-06456-0 (PMC10155408; doi:10.1186/s12891-023-06456-0)
Supplement: Supplementary file 1 — Supplementary Material 1 [file 12891_2023_6456_MOESM1_ESM.docx]

**The Royal Bournemouth Hospital**

Castle Lane East
Bournemouth
Dorset
BH7 7DW

*[Date]*

*[Participant’s Name]*

*[Participant’s Address]*

Dear *[Participant’s Name]*

**Study Title: A pragmatic multi-centered randomised controlled trial with economic evaluation, to compare a cycling and educational programme with usual physiotherapy care in the treatment of hip osteoarthritis: CycLing and EducATion (CLEAT)**

We are writing to you as you have been referred to physiotherapy for treatment for hip pain or hip osteoarthritis and we would like to offer you the opportunity to consider taking part in a research study.

This study has been designed to assess whether there is an improvement in hip function and pain between patients receiving cycling and education compared to those receiving standard physiotherapy care. More information is provided in the Patient Information Sheet included with this letter.

We would be grateful if you could contact [*Name*] on our study team by telephone: [*number*] or email [*address*] to let them know whether you are interested in taking part in CLEAT. Please also don’t hesitate to get in contact if you have any questions about the study.

We will follow-up with a quick phone call if we have not heard from you within the next two weeks.

Your participation is entirely voluntary; you are under no obligation to take part in this study. Your decision to take part or not will not affect your care in any way.

Yours sincerely

Professor Tom Wainwright PhD PgDip PgCert BSc (Hons) MCSP

Clinical Researcher in Orthopaedics

Professor in Orthopaedics (Bournemouth University)

Enclosed: Participant information sheet; Consent Form (for completion at visit)
