# Supplementary material for: CycLing and EducATion (CLEAT): protocol for a single centre randomised controlled trial of a cycling and education intervention versus standard physiotherapy care for the treatment of hip osteoarthritis
Source: BMC Musculoskelet Disord. 2023 May 3;24:344. doi: 10.1186/s12891-023-06456-0 (PMC10155408; doi:10.1186/s12891-023-06456-0)
Supplement: Supplementary file 2 — Supplementary Material 2 [file 12891_2023_6456_MOESM2_ESM.docx]

**CLEAT: A Study to Compare Physiotherapy Treatment with a Static Cycling and Education Programme to Help Improve Hip Pain**

We would like to invite you to take part in a research study

- Before you decide we would like you to understand why the research is being done, and what it would involve for you.
- Please take time to read the following information and discuss with family and friends if you wish.
- **A member of the research team will go through this information sheet with you and answer any questions you may have.**
- You are free to decide whether or not to take part in this trial. If you choose not to take part, this will not affect the care you receive from your doctors.

Important things that you need to know

- We want to find the best way to treat patients who have hip pain associated with Hip Osteoarthritis
- We are comparing standard physiotherapy treatment that is offered by the NHS with an 8 week group static cycling and education programme.
- You have been invited to participate in this study because you have been referred to receive physiotherapy treatment for your hip pain.
- If you decide to take part in this study you will need to attend a pre-treatment assessment at [*the Orthopaedic Institute (ORI) at Bournemouth University or other suitable facility*] and then another assessment after your 8 week treatment has completed. We will also ask you to complete a questionnaire which will be sent to you 6 months after your first assessment.
- If you agree to participate, you will be one of approximately 250 individuals who will be taking part in this research study across the Wessex region.
- You can stop taking part in the study at any time

Contents

**P2-6 Part 1**

Why are we doing the study?

Do I have to take part?

What will I need to do if I agree to take part?

What are the possible disadvantages and risks? What are the possible benefits?

Will my taking part in the study be kept confidential?

**P6-9 Part 2**

More information on taking part

**P9** How to contact us


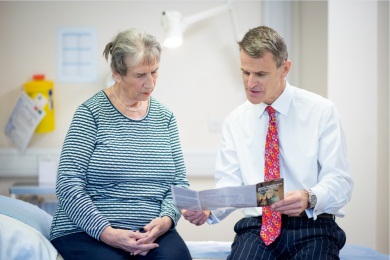


How to contact us

If you have any questions about this study, please talk to the physiotherapist who has organised it Tom Wainwright on **01202 961656** or the trial manager [*Name*] on [***contact number*].**

**PART 1** tells you the purpose of this study and what will happen to you if you take part.

**What is the purpose of the study?**

The purpose of this study is to look at whether people who receive an 8 week education and static cycling treatment have an improvement in their hip function and less pain than those patients who receive physiotherapy treatment as per standard care and practice within the NHS.

There is no known cure for osteoarthritis, and the latest National Institute for Health and Care Excellence (NICE) guidelines for people with symptoms not yet severe enough for surgery recommend education and advice, exercise (including local muscle strengthening and aerobic exercise) and weight loss, if needed, and so it is important to develop these strategies further to support patients.

Cycling may be of benefit in comparison to other forms of exercise because it is a healthy form of physical activity, is a non-weight bearing activity that is considered less stressful on the body than impact or other running sports, and has been shown to improve muscle weakness.

In this study, half of the patients will receive the standard physiotherapy treatment that is available through the NHS. The other half of the patients will receive weekly group education and static cycling sessions designed to increase strength and function through physical activity. Whether you receive standard physiotherapy care or the weekly static cycling and education sessions will be decided at random, similar to the toss of a coin and your treatment will be provided over the course of 8 weeks, with an assessment taken before and after your 8 week treatment block.

The results of this study will be based on your opinions with regard to changes in pain, ability to perform everyday activities, and quality of life, and from information collected by the research team from the assessments taken before and after your treatment and from a final questionnaire sent to you 6 months following your first pre-study assessment.

**Do I have to take part?**

No, it is entirely up to you to decide whether or not you would like to take part. Please keep this information sheet and use it to make your decision. If you decide you would like to know more about the study you will be invited to talk to a member of the research team who will describe the study in more detail and go through this information sheet.

If you agree to participate you will be asked to sign a consent form. You are free to withdraw at any time, without giving a reason. This will not affect the standard of care you receive. Should you choose not to participate you will receive the standard NHS treatment for your condition.

**What will happen to me if I agree to take part?**

If you decide you would like to take part you will receive a phone call from one of the study team to discuss the study and any questions that you have. If you are satisfied that all of your questions have been answered and agree to take part in the study they will ask you to verbally consent to participate. Once you have consented you will be asked some study related questions about your quality of life and be invited to the [*Orthopaedic Research Institute (ORI), Bournemouth University, or other suitable facility*](parking will be provided) for your baseline visit, where you will need to sign an informed consent form before undertaking assessments.

At the visit we will measure your general health including height, weight, blood pressure, heart rate and you will need to do some static cycling to assess your suitability for the study. You will be asked to complete some questionnaires about your pain, the effect on your quality of life and your ability to perform day-to-day tasks, such as climbing the stairs, getting in and out of the car and carrying shopping. We will measure how easily you can move your affected hip, the number of times you can move from sitting to standing in 30 seconds, the time it takes for you to walk for 40 metres, and climb some stairs. We anticipate that your baseline visit will take approximately 45 minutes.


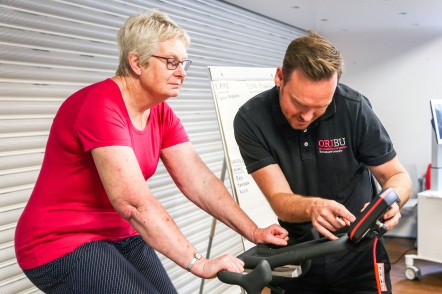
 We may need to review your medical records for relevant previous medical history to ensure that there is no reason that you cannot take part in the study. Access to your medical records will be provided by the NHS Physiotherapy Clinic from which you were referred to the authorised members of the research team at the University Hospitals Dorset NHS Foundation Trust.

After this a member of the study team will enter your details into a computer program which will make a decision about which group you will be in whilst you are in the study. This allocation is made by chance, similar to the toss of a coin. This is important because it ensures that the treatments are tested fairly and no one can guess the group the computer puts you into.

The computer will allocate you to either standard physiotherapy treatment consisting of up to four 30 minute appointments over 8 weeks or to the education and static cycling group where you will attend weekly 1 hour group sessions consisting of 30 minutes of education led by a trained physiotherapist, followed by 30 minutes of static cycling with a qualified instructor every week for 8 weeks.

Education sessions will consist of a short 5 minute video followed by a general discussion of some of the points highlighted in the video. Each week we will cover different themes related to osteoarthritis including benefits of exercise, types of pain relief, complementary therapies, diet and nutrition.

Cycling sessions will be designed with varying speeds and intensity, building week on week to ensure a steady progress for each person. For example, week 1 will start with bike set up and gentle cycling through to week 8 where you will participate in a 35 to 40 minute cycle equivalent to a standard spin class, at an intensity that you are able to comfortably sustain.

You will be given a home exercise programme as part of your treatment. We will also ask you to keep an activity diary during the 8 weeks of treatment to track any activity you take part in, including prescribed home exercises, outside of your physiotherapy or education and cycling sessions. These activity diaries will be reviewed by the physiotherapists involved in your treatment on each treatment visit.

Approximately 2 weeks after your treatment ends you will be asked to return to [*ORI, or other suitable facility*] to repeat the tests and questionnaires you participated in before your treatment. Six months from the start of your treatment we will send you (either by post or email) questionnaires similar to the ones you would have completed at previous appointments. These will be used to see the difference your treatment sessions have made. You will receive a phone call from one of the study team so that these questionnaires can be completed over the telephone. If you are not available, we will send them to you and ask you to complete them and return back to the Trial Manager in a pre-paid envelope. Once the questionnaires have been completed and returned at 6 months that is the end of the study and you will not be required to do anything study specific after this time.

If you are randomised to the education and static cycling group, as part of this treatment we will send you a report showing how your assessment scores after the group sessions have changed from those measured before. This report will also go to your GP. If you are randomised to standard physiotherapy care your GP will be contacted by the physiotherapist as per routine practice.

At all visits, you will be asked about any difficulties you may have experienced since your last visit, including any hospital admissions, visits to your GP and if you have changed your medication.

Any other on-going care/treatments that you require will not be affected by your participation in this study.

**What will I have to do?**

If you are currently participating in another clinical trial (or have recently participated) then please inform the research team so they can make sure that this does not stop us from enrolling you in this study.

It is important that you attend your follow-up appointment for this study. We will also need you to remember or record any illnesses that you have experienced whilst undergoing treatment on the study, for which you needed to see a health practitioner, and any change in medication or treatment that you receive.

**Expenses and Payments**

You will not be paid for your participation in this study. You will not incur any expenses above that of standard care.

All ongoing care that might be required will be provided by the NHS.

**What are the alternatives for treatment?**

If you decide not to take part in this study, you will remain within the referral list for standard NHS physiotherapy treatment. Whether you decide to take part or not, there will be no delay to your treatment.

**What are the possible disadvantages and risks of taking part?**

There are only minimal risks involved in this research.

You will be asked to cycle on a static bike if you are selected to take part in the education and cycling sessions. There is a possible risk of feeling a little sore after exercising or being more active, however you will be guided by your physiotherapist and by the cycling instructor whilst you are cycling in the classes and will be able to seek their opinions about joint and muscle soreness. During the education sessions there will be some group discussions; although you are encouraged to participate in these group discussions, you will not be asked to share any information that you do not wish to, and if you feel distressed in any way you will be supported by the physiotherapist leading the group.


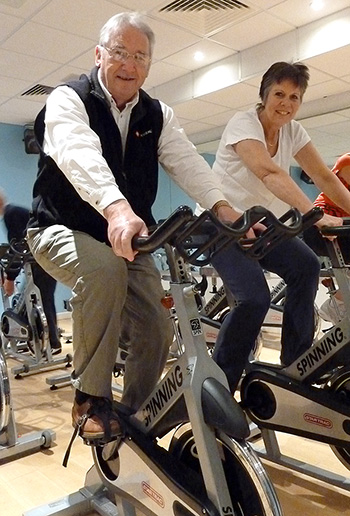
Occasionally people feel uncomfortable answering certain questions about their health. If you are asked questions that you feel uncomfortable with by the researcher, physiotherapist or within the questionnaires, then you do not have to answer them.

**For women**

If you are pregnant or planning to be pregnant you will still be eligible for the study providing you already take part in regular exercise equivalent to at least 30 minutes of cycling once a week.

**What are the possible benefits of taking part?**

We cannot promise the study will help you but we hope that the information we obtain from this study will help improve treatment for patients with hip osteoarthritis in the future, and will find out if cycling can help improve hip function and pain. If the study shows that cycling can have a positive effect on patients with this condition then this might be made to be a part of the treatment process in the future and will help patients across the country.

By taking part in this research you will help us to understand more about activity levels in patients with hip osteoarthritis so that we can improve treatment for similar patients in the future.

There may not be any benefit to you in taking part in this study; however research like this helps to continually improve the treatments and care provided to all patients now and in the future by collecting information on what does and doesn’t help.

**Will my taking part in the study be kept confidential?**

Yes. We will follow ethical and legal practice and all information about you will be handled in confidence. The details are included in Part 2.

**This completes part 1.**

If the information in Part 1 has interested you and you are considering participation, please read the additional information in Part 2 before making any decision.

**PART 2** gives you more detailed information about the conduct of the study.

**What if relevant new information becomes available?**

Sometimes we get new information which may be relevant to this study. If this happens, a member of the study team will discuss this information with you and discuss whether you should continue and alternative options/treatment if appropriate. If you decide not to continue on the study, we will make arrangements for your care to continue normally.

If the study is stopped for any other reason, we will tell you and arrange your continuing care.

**What will happen if I don’t want to carry on with the study?**

You can withdraw from participation in the study at any time without giving a reason. If you withdraw we will use all of the information that we have collected about you up to that point.

**What if there is a problem?**

If you have a concern about any aspect of this study, you should ask to speak to the researchers who will do their best to answer your questions or [*Trial Manager*] on [*contact number*]**.** If you remain unhappy and wish to complain formally, you can do this through the hospital complaints procedure. Details can be obtained from the hospital Patient Advice and Liaison Services (PALS) on [*phone number*].

**Harm**

In the event that something does go wrong and you are harmed during the study and this harm is due to someone‘s negligence then you may have grounds for legal action for compensation. The normal National Health Service complaints mechanisms will be available to you (if appropriate).

**How will we use information about you?**

We will need to use information from you for this research project. This information will include your:

- Initials
- NHS Number
- Name
- Contact Details
- Gender
- Date of birth
- Ethnicity
- Education
- Current employment status

People will use this information to do the research or to check your records to make sure that the research is being done properly. People who do not need to know who you are will not be able to see your name or contact details. Your data will have a code number instead. We keep all information about you safe and secure.

Once we have finished the study, we will keep some of the data so we can check the results. We will write our reports in a way that no-one can work out that you took part in the study.

**What are your choices about how your information is used?**

- You can stop being part of the study at any time, without giving a reason, but we will keep information about you that we already have.
- We need to manage your records in specific ways for the research to be reliable. This means that we won’t be able to let you see or change the data that we hold about you.

**Where can you find out more about how your information is used?**

You can find out more about how we use your information

- at [www.hra.nhs.uk/information-about-patients/](https://www.hra.nhs.uk/information-about-patients/)
- on our research website <https://intranet.rbch.nhs.uk/index.php/research>
- by asking one of the research team
- by sending an email to [Information.Governance@uhd.nhs.uk](mailto:Information.Governance@uhd.nhs.uk),
- or by ringing us on 0300 019 4461.

University Hospitals Dorset (UHD) will keep identifiable information about you from this study for a minimum of 5 years after the study has finished.

**What will happen to the results of the research study?**

The results will be published in relevant scientific journals and presented at conferences and meetings. No personal identifiable data will be disclosed in any publication. The results from the study will also be made available on the Orthopaedic Research Institute’s website <https://microsites.bournemouth.ac.uk/ori/> and the UHD’s Research and Innovation (R&I) website.

**Who is organising and funding the research?**

The National Institute for Health Research (NIHR) – Research for Patient Benefit, will provide your hospital with reimbursement for including you in this study. This payment will be to cover staff costs and time for including you in the study.

**Who has reviewed the study?**

All research is looked at by an independent group of people called a Research Ethics Committee, to protect your interests. This study has been reviewed and given favourable opinion by the South Central Oxford C Research Ethics Committee and the Health Research Authority and likewise has been reviewed by Bournemouth University’s Science, Technology & Health Research Ethics Panel.

You will be given a copy of this information sheet and the signed consent form to keep.

**Further information and contact details**

You may ask any questions you like at any time about your rights as a participant in a research study, or about the research study itself. Your physiotherapist will be available to discuss these issues with you in clinic, or the Trial Manager at your pre-study assessment, alternatively you can contact the Trial Manager [*name*] if you phone them on [*phone number*].

You may also contact Patient Advice and Liaison Services (PALS): [pals@uhd.nhs.uk](mailto:pals@uhd.nhs.uk) or 0300 019 4886 if you would like some independent advice before entering the study.

Thank you for taking the time to read this information sheet.
