# Supplementary material for: CycLing and EducATion (CLEAT): protocol for a single centre randomised controlled trial of a cycling and education intervention versus standard physiotherapy care for the treatment of hip osteoarthritis
Source: BMC Musculoskelet Disord. 2023 May 3;24:344. doi: 10.1186/s12891-023-06456-0 (PMC10155408; doi:10.1186/s12891-023-06456-0)
Supplement: Supplementary file 3 — Supplementary Material 3 [file 12891_2023_6456_MOESM3_ESM.docx]

**INFORMED CONSENT FORM**

**STUDY**:  **CLEAT: Cycling and Education against Hip Pain.**

**REC No: 19/SC/0502**

**Chief Investigator: Thomas Wainwright Site No: 1 Patient ID No: ………..**

Please initial box

1. I confirm that I have read the information sheet dated 01.06.21 v2.2 for the above study and
   I have had the opportunity to consider the information, ask questions and have had these
   answered satisfactorily.
2. I understand that my participation is voluntary and that I am free to withdraw at any time
   without giving any reason, without my medical care or legal rights being affected.
3. I understand that relevant sections of my medical notes and data collected during
   the study may be looked at by individuals from regulatory authorities or from the NHS
   Trust, or employed by the sponsor where it is relevant to my taking part in this research.

I give permission for these individuals to have access to my records.

YES NO

1. I consent to my GP being made aware of the results of the study procedures.
2. I have received an explanation of the nature, purpose, duration of the personal data processing and was able to question about it.
3. I understand that while findings from the study may be published, I will not be identified

and my personal results will remain confidential.

1. I understand that my data may be included in an anonymised form within a dataset to be archived at Bournemouth University’s Online Research Data Repository, BORDaR.
2. If I decide to withdraw from the study, I consent to allowing access to my medical records

for the duration of the research in order for my outcome data to be used in the final analysis.

1. I agree to take part in the above study.
2. I agree that my contact information is kept in a secure database after the trial has ended so that I may be contacted about any other relevant clinical trials in the future.

Name of Participant Date Signature

Name of Person receiving consent Date Signature

**When completed: 1 for participant; 1 for researcher site file; 1 (original) to be kept in medical notes.**
