# Supplementary material for: CycLing and EducATion (CLEAT): protocol for a single centre randomised controlled trial of a cycling and education intervention versus standard physiotherapy care for the treatment of hip osteoarthritis
Source: BMC Musculoskelet Disord. 2023 May 3;24:344. doi: 10.1186/s12891-023-06456-0 (PMC10155408; doi:10.1186/s12891-023-06456-0)
Supplement: Supplementary file 5 — Supplementary Material 5 [file 12891_2023_6456_MOESM5_ESM.docx]

| Study: | **CLEAT Trial** | IRAS: | | | **232991** | | PI: | | **T Wainwright** | Ext: |  |
| --- | --- | --- | --- | --- | --- | --- | --- | --- | --- | --- | --- |
| Patient ID: |  | Patient Initials: | | |  | | Visit: | | **Physiotherapy** | Date: |  |
| **RESEARCH – DO NOT DESTROY** | | | | | | | | | | | |
| **PROFORMA – PHYSIOTHERAPY Telephone  Face to Face** | | | | | | | | | | | |
|  | | | | | |  | |  | | | |
| **PHYSIOTHERAPY SESSION** | | | | | |  | | (*Please write in the box which appointment number this is for this participant i.e 1, 2, 3 or 4*) | | | |
|  | | | | | |  | |  | | | |
|  | | | | | |  | |  | | | |
| \| **Treatment Type** \| **Yes/No** \| **Details of Treatment**  ***(tick as many as applies to this appointment)*** \| **Duration**  ***(in total, not per individual option)*** \| **Dose/Intensity *(use Borg Scale shown over page)*** \| \| --- \| --- \| --- \| --- \| --- \| \| Education/Advice \| **YES  NO** \| \| Osteoarthritis \|  \| \| --- \| --- \| \| Pain Relief \|  \| \| Pacing \|  \| \| Diet/nutrition/weight loss \|  \| \| Supplements \|  \| \| Assistive devices and footwear \|  \| \| Surgery \|  \| \|  \| N/A \| \| (min) \| \| Cardiovascular Exercise \| **YES  NO** \| \| Cycling \|  \| \| --- \| --- \| \| Walking \|  \| \| Running \|  \| \| Swimming \|  \| \| Rower \|  \| \| Stepper \|  \| \| Cross Trainer \|  \| \| Other \|  \| \| Click here to enter text. \| Click here to enter text. \| \| (min) \| Borg \| \| Strength Exercise \| **YES  NO** \| \| Body weight exercise \|  \| \| --- \| --- \| \| Weighted exercise \|  \| \|  \|  \| \| (min) \| Borg \| \| Stretch \| **YES  NO** \| \| Static \|  \| \| --- \| --- \| \| Dynamic \|  \| \| Click here to enter text. \| Click here to enter text. \| \| (min) \| Borg \| \| Balance/Proprioception \| **YES  NO** \| \| Static \|  \| \| --- \| --- \| \| Dynamic \|  \| \|  \|  \| \| (min) \| Borg \| \| Manual Therapy \| **YES  NO** \| \| Massage \|  \| \| --- \| --- \| \| Mobilisation \|  \| \| Manipulation \|  \| \| Click here to enter text. \| N/A \| \| (min) \| | | | | | | | | | | | |
| **Table continued over page…..** | | | | | | | | | | | |
| **….Table continued from previous page** | | | | | | | | | | | |
|  | | | | | | | | | | | |
| \| **Treatment Type** \| **Yes/No** \| **Details of Treatment**  ***(tick as many as applies to this appointment)*** \| **Duration**  ***(in total, not per individual option)*** \| **Dose/Intensity *(use Borg Scale shown below)*** \| \| --- \| --- \| --- \| --- \| --- \| \| Electro-physical Treatment \| **YES  NO** \| \| Ultrasound \|  \| \| --- \| --- \| \| PSWT* \|  \| \| Shockwave \|  \| \| Other \|  \| \| Click here to enter text. \| N/A \| \| (min) \| \| Thermo-Therapy \| **YES  NO** \| \| Ice \|  \| \| --- \| --- \| \| Heat \|  \| \| Click here to enter text. \| N/A \| \| (min) \| \| Acupuncture \| **YES  NO** \| ***DO NOT WRITE ANYTHING HERE*** \| Click here to enter text. \| N/A \| \| (min) \| \| Orthotics/Bracing \| **YES  NO** \| \| Brace \|  \| \| --- \| --- \| \| Orthotic \|  \| \| Click here to enter text. \| N/A \| \| (days/ permanent) \| | | | | | | | | | | | |
| *PES = Pulsed Shortwave Therapy | | | | | | | | | | | |
| 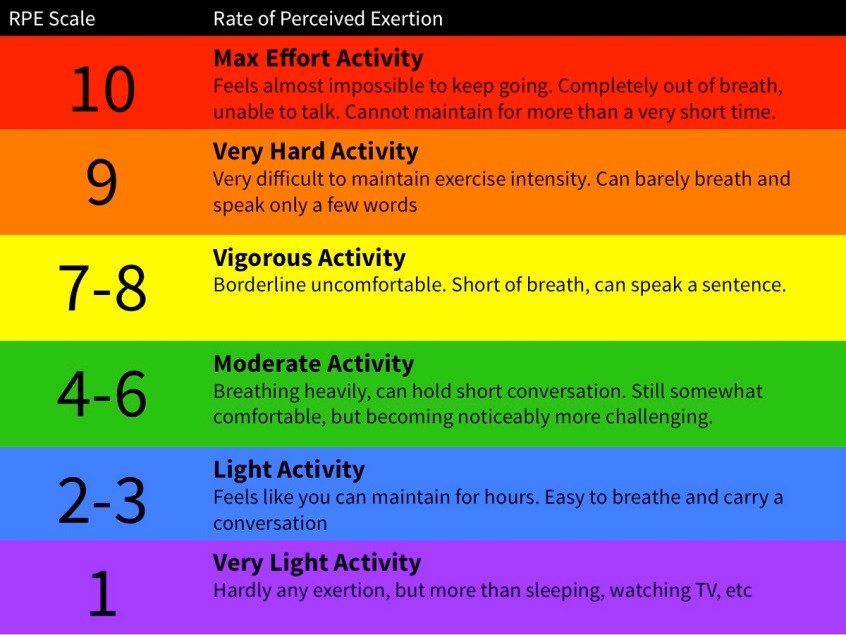    The next appointment is booked for (date) ________________________  ***OR please tick box if patient discharged from physiotherapy*** | | | | | | | | | | | |
| Completed By  *Full Name* | | | |  | | | | | | | |
| Signed | | |  | | | | Date | | |  | |
